# Differential Item Functioning of informant measures of cognitive functioning in the U.S. and Mexico

**DOI:** 10.1002/alz70860_107153

**Published:** 2025-12-23

**Authors:** Phillip A Cantu, Jocelyn Jaen, Mark E. Sanderson‐Cimino, Douglas Tommet, Keith F. Widaman, Rich Jones, Alden L. Gross

**Affiliations:** ^1^ UTMB, Galveston, TX, USA; ^2^ Rush Alzheimer's Disease Center, Chicago, IL, USA; ^3^ Memory and Aging Center, UCSF Weill Institute for Neurosciences, University of California, San Francisco, San Francisco, CA, USA; ^4^ Brown University School of Medicine, Providence, RI, USA; ^5^ University of California at Riverside, Riverside, CA, USA; ^6^ Brown University, Providence, RI, USA; ^7^ Center on Aging and Health, Johns Hopkins University, Baltimore, MD, USA

## Abstract

**Background:**

Population studies of cognitive functioning rely on survey questionnaires to estimate the prevalence of dementia in nationally representative samples. In addition to direct tests of cognitive functioning, the international studies as part of the Harmonized Cognitive Assessment Protocol, also include informant questionnaires, in which a family member or other person close to the research participant are asked about the participant's cognitive functioning. While international harmonized measures of directly assessed cognitive have been developed, there are no statistical harmonizations of informant assessments of cognitive functioning.

**Method:**

This study investigates differences in how informants respond to questions about cognitive functioning in the US and Mexico. Using data from the Harmonized Cognitive Assessment Protocol (HCAP) of the Health and Retirement Study (HRS) (*n* = 3,183) in the United States and the Mexican Cognitive Aging Study (MexCOG) (*n* = 1,259) in Mexico, we examined informant reports of cognitive functioning of the Community Screening Instrument for Dementia (CSID). We test for differential item functioning (DIF) using structural equation modeling (SEM) to develop a confirmatory factor analysis (CFA) model of the CSID informant questionnaire in Mexico and the U.S.

**Result:**

We find that a harmonized measure of the CSID has fixed factor loadings between countries but different item thresholds. Our harmonized measure of informant reports of cognitive functioning suggests that informants report less cognitive impairment for older adults in Mexico when controlling for directly assessed cognitive functioning.

**Conclusion:**

Our results suggests that either the harmonized direct assessment of cognition may underestimate cognitive functioning in Mexico or that informants in Mexico are systematically under reporting symptoms of cognitive decline.

